# Group Care in the first 1000 days: implementation and process evaluation of contextually adapted antenatal and postnatal group care targeting diverse vulnerable populations in high-, middle- and low-resource settings

**DOI:** 10.1186/s43058-022-00370-7

**Published:** 2022-11-24

**Authors:** Nele Martens, Mathilde R. Crone, Ashna Hindori-Mohangoo, Manodj Hindori, Ria Reis, Ilir S. Hoxha, Jedidia Abanga, Shanaaz Matthews, Lizette Berry, Rianne M. J. J. van der Kleij, M. Elske van den Akker-van Marle, Astrid van Damme, Florence Talrich, Katrien Beeckman, Christine Mc Court, Sharon Schindler Rising, Deborah L. Billings, Marlies Rijnders

**Affiliations:** 1 Leiden University Medical Centre, Leiden, Netherlands; 2 Foundation for Perinatal Interventions and Research in Suriname, Paramaribo, Suriname; 3 Amsterdam Institute for Global Health and Development (AIGHD), Amsterdam, Netherlands; 4 University of Cape Town, Cape Town, South Africa; 5 Evidence Synthesis Group Prishtina, Prishtina, Republic of Kosovo; 6 Action for Mothers and Children, Prishtina, Republic of Kosovo; 7 Presby Health Services, Accra, Ghana; 8 Vrije Universiteit Brussel (VUB), Universitair ziekenhuis Brussel (UZ Brussel), Brussels, Belgium; 9 Universiteit Antwerpen, Antwerp, Belgium; 10 City University of London, London, UK; 11 Group Care Global, Philadelphia, USA; 12 Netherlands Organisation for Applied Scientific Research, The Hague, Netherlands

**Keywords:** Antenatal group care, Postnatal group care, Implementation, Realist evaluation, Vulnerable populations, Contextual adaptation, Consolidated Framework for Implementation Research (CFIR)

## Abstract

**Background:**

Group care (GC) improves the quality of maternity care, stimulates women’s participation in their own care and facilitates growth of women’s social support networks. There is an urgent need to identify and disseminate the best mechanisms for implementing GC in ways that are feasible, context appropriate and sustainable. This protocol presents the aims and methods of an innovative implementation research project entitled *Group Care in the first 1000 days (GC_1000)*, which addresses this need.

**Aims:**

The aim of GC_1000 is to co-create and disseminate evidence-based implementation strategies and tools to support successful implementation and scale-up of GC in health systems throughout the world, with particular attention to the needs of ‘vulnerable’ populations.

**Methods:**

By working through five inter-related work packages, each with specific tasks, objectives and deliverables, the global research team will systematically examine and document the implementation and scale-up processes of antenatal and postnatal GC in seven different countries. The GC_1000 project is grounded theoretically in the consolidated framework for implementation research (CFIR), while the process evaluation is guided by ‘Realistic Evaluation’ principles. Data are gathered across all research phases and analysis at each stage is synthesized to develop Context-Intervention-Mechanism-Outcome configurations.

**Discussion:**

GC_1000 will generate evidence-based knowledge about the integration of complex interventions into diverse health care systems. The 4-year project also will pave the way for sustained implementation of GC, significantly benefitting populations with adverse pregnancy and birthing experiences as well as poor outcomes.

Contributions to the literature

- Contributions to the sparse literature on theory-informed implementation research in maternal-child health care.
- A toolbox for the adaptation, implementation and scale up of group antenatal and postnatal+ care, based on findings on implementation process, model fidelity, sustainability, costs, indicators of impact and perceptions of benefit.
- Contextually driven implementation strategies and adaptations to the group care model through the application of realist evaluation principles.

## Background

Despite vast improvements over the past two decades, adverse maternal and neonatal outcomes remain major challenges today. This is not only reflected in global health data but also in the United Nations’ Sustainable Development Goals (SDGs), which stress the need to improve reproductive, maternal, new born and child health [1]. Despite a 38% decline in the maternal mortality ratio (MMR) since the year 2000 [2], still too many mothers and babies die during pregnancy, labour and postpartum. In 2017, 295,000 women died worldwide due to pregnancy complications or childbirth [2]. This translates to an average of 810 women per day dying from preventable causes related to pregnancy and childbirth. Although 94% of these deaths occurred in low- and middle-income countries (LMIC) [2], poor pregnancy outcomes have also been reported amongst so called vulnerable^1^ groups in high-income countries [4, 5]. The main causes of maternal death are severe bleeding, infections, pre-eclampsia and eclampsia, birth complications and unsafe abortions [6]. In most cases, these conditions can be addressed and do not need to be fatal when recognized in a timely manner [7].

**Fig. 1:**
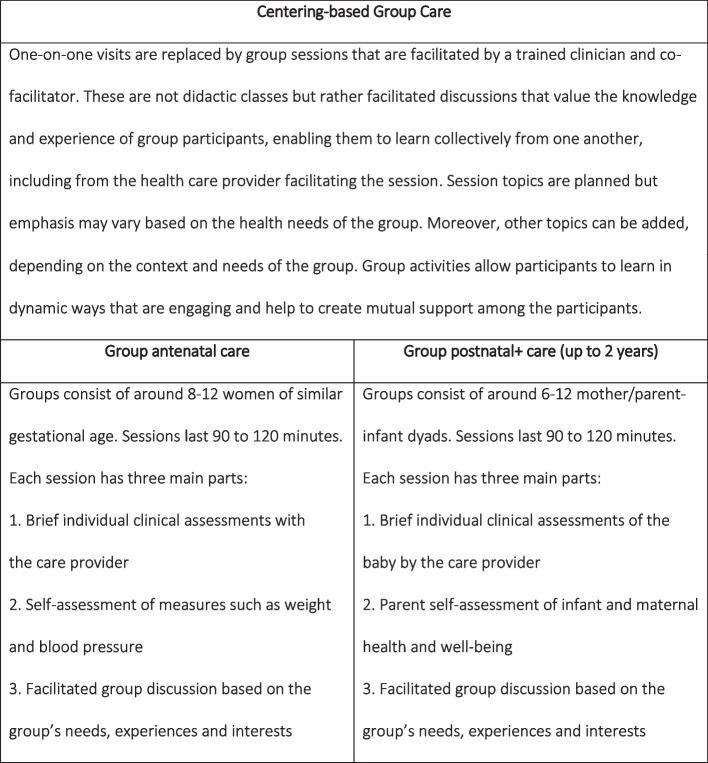
The Centering-based Group Care model

Newborns also are at particular risk during child birth and the postpartum period. In 2019, 2.4 million babies died in their first month of life [8]. While children are at greatest risk of death during the first 28 days after birth [8], the first years of life lay the foundation for physical and mental well-being from infancy to and throughout adulthood [9]. Thus, accessible and high-quality antenatal and postnatal care are not only a human right [10], but together they can build the basis for healthy development over the life span [11]. While this has the potential to ultimately foster a healthy population and reduce health expenses in the long-term, access to high-quality maternal health care services remains a privilege. Key factors preventing women from receiving appropriate care include poverty, distance to facilities, lack of information, harmful cultural beliefs and practices and poor quality, disrespectful, or lack of humanized care [7, 12]. Poor quality of services often results from shortage of staff and resources, as well as hierarchies and power dimensions within health care and an inattention to human rights [6, 7, 12],

.In order to improve the quality of maternity care and to stimulate women’s participation in their own care, a practising midwife developed ‘group care’ (GC) for antenatal care in the early 90s. Postnatal group/parenting care was subsequently developed so that a continuum of care was available to parents [13]. GC can help to break the vicious cycle of poor quality and inadequate utilization of services by offering care that addresses health holistically, with an integrated approach to health assessment, health education and support. Centering-based GC (CBGC) is a model that was first developed in the USA, consisting of three core components: (1) health care in the form of self-assessments by women and parents, and individual health check-ups conducted by trained clinicians; (2) interactive learning; and (3) peer support/community building [13, 14]. Figure 1 describes the CBGC model in more detail. Whereas educative pregnancy group programmes organized outside of routine care are likely to be attended by mothers of mostly high social economic status, CBGC is explicitly offered in and as part of routine care, which makes it accessible to *all* mothers/parents. Moreover, as CBGC is not merely an educational programme but it also contains a health care component, it can replace routine one-on-one care.

While no direct impact on maternal mortality and infant survival have been demonstrated, improved birth outcomes, such as higher birth-weight and lower preterm-birth rates, have been reported amongst women who attended antenatal GC [15–19]. In two studies, preterm birth rates were particularly reduced for low-income African-American women participating in GC in the USA, which suggests that marginalized or under-served populations can benefit from GC [16, 17]. However, according to recent systematic reviews, the evidence is still not sufficient to unconditionally claim that CBGC leads to improved birth outcomes [20–22]. Even if CBGC did not significantly ameliorate the rates of preterm birth and low birth weight, the most recent review, including only randomized controlled trials, reports that the overall rates of preterm birth and low birth weight were lower in CBGC groups compared to individual care. In addition, it showed some evidence for improved psychosocial outcomes in CBGC-groups.

Other important benefits of CBGC, described in qualitative research, include an improved woman-provider experience, enhanced self-care, empowerment, enhanced learning about health behaviours, enriched networks of relationships and increased social support [23]. CBGC has also been shown to raise clinicians’ motivation [24–27] and may provide savings to the health care system [28, 29]. Moreover, antenatal CBGC has been shown in some settings to increase women’s attendance at antenatal and postnatal visits significantly. For example, one study in Malawi and Tanzania showed that 94% of women in antenatal CBGC versus 58% in individual care attended all recommended ANC visits and 75% versus 50% attended the 6-week postnatal visit [30]. Despite these promising findings, the CBGC model has not been integrated into standard midwifery/obstetric or maternity care outside the USA and disparate factors are likely to impact the implementation of CBGC in diverse health care systems. Frequently implementation, i.e. the act of carrying out an intention into effect [31], fails when contextual factors are not considered [32, 33]; implementation failure can be mitigated by developing and applying contextually driven implementation strategies [34–37].

This article presents the aims and methods of an innovative implementation research project entitled *GC during the first 1000 days (GC_1000)*, which addresses the need to identify and disseminate the best mechanisms for implementing GC in ways that are feasible, appropriate to context, sustainable and scalable. GC_1000 began in January 2020 and is funded for a four-year period through the European Commission’s Horizon 2020 research and innovation programme under grant agreement 848147.

### Aims

The overall aim of GC_1000 is to co-create and disseminate evidence-based implementation strategies and tools to support successful implementation and scale-up of GC in the first 1000 days in health systems throughout the world, with particular attention to the needs of vulnerable populations. The project takes place in seven countries and has five specific objectives:

1. To identify context-specific factors that enhance or impede transition from individual provider-to-user care to antenatal and postnatal+ group care, considering the needs of women and families, the issues care providers face and the opportunities and restrictions of health care systems
2. To develop and employ implementation strategies adapted to the specific contextual needs, leading to successful implementation of GC with at least five antenatal and/or postnatal+ groups per country
3. To monitor and evaluate the implementation of GC regarding process, fidelity, sustainability, costs, indicators of impact and perceptions of benefit
4. To develop and deliver seven country blueprints for the scale-up of antenatal and postnatal GC based on implementation success and challenges
5. To develop and disseminate a GC_1000 implementation strategy toolbox for the adaptation, implementation and scale up of group antenatal and postnatal+ care

## Methods/design

*Implementation sites* are located in seven countries including four European (The Netherlands, Belgium, England and Kosovo), two African (Ghana and South Africa) and one South American (Suriname) (Table 1). This selection of countries allows for capturing diversity with regard to implementation challenges, health systems and cultural and economic factors, which will ultimately enable the development of a widely applicable implementation strategy toolbox (Table 1).

**Table 1:** Implementing countries and their rationale for inclusion

Country	Rationale for inclusion
Suriname	Suriname has high rates of maternal deaths (MMR of 120 per 100,000 live births) and perinatal deaths (25 per 1000 births) and adverse birth outcomes. Adverse pregnancy and birth outcomes have been associated with socio-demographics and environmental factors, such as lack of social support, insufficient knowledge, poor living conditions and substandard care. Antenatal GC was introduced in Suriname in 2014 as the SamenZwanger-health care model and its expansion can help to improve maternal and child health in Suriname. As such, the GC model has to be adapted for vulnerable women and it will be implemented in deprived communities.
The Netherlands	In the Netherlands, the number of adverse perinatal outcomes is higher in non-Western women and in Western women living in disadvantaged areas. Adverse outcomes are associated with lifestyle but also with system failure. It has been argued that specific care and attention should be given to so-called marginalized groups and recently the government funded the programme ‘A promising Start’ aimed at addressing health inequalities during the first 1000 days of child’s life. Although group ANC has been successfully implemented, it needs to be expanded to mother-infant care and adapted to better reach under-served, marginalized and migrant women.
England	A government recommendation in 2010 highlighted the priority to early infant years including maternal and infant health to achieve a long-term sustainable reduction in health inequalities. English policy for maternity services in 2015, Better Births, recommended a greater focus on continuity of carer, personalized care and attention to perinatal mental health. Currently, a model of group antenatal, Pregnancy Circles, tailored to a local community and services in an inner-city area of high socio-economic, cultural, ethnic and linguistic diversity is being researched. The model will be further researched and expanded to postnatal care.
Ghana	Access to quality of health services is still challenging for rural communities in Northern Ghana. For instance, while it takes an average 30 min to reach a health facility in urban Ghana, in some parts of rural Northern Ghana accessing a health facility can take as much 3 h. There is a lack of adequate testing materials for ANC in most rural facilities. Psycho-social care, birth preparedness plans and parenting information are not adequately covered during antenatal and postnatal visits. It is anticipated that antenatal GC services tailored to women’s needs will be delivered to rural and poor communicates in Ghana.
Kosovo	The infant mortality rate in Kosovo is the highest in Europe. One of the major challenges is to improve parenting skills as a lack of knowledge about adequate home care management, child physical and cognitive development and reproductive health prevails. Further, the immunization rate remains low amongst Roma, Ashkali and Egyptian communities and inappropriate breastfeeding and infant feeding patterns raise major concerns. Most women do not receive any preventative educational services; hence, system change towards Group antenatal and mother-infant care in Kosovo can strengthen the provision of women-centred care that is informative, supportive and empowering especially for the underserved Roma population.
Belgium	Large cities in Belgium are characterized by high levels of poverty. In Brussels, 33% of the children are born in poverty. Inequities in health care have been identified as evidenced by an increased perinatal mortality rate amongst children of mothers with low educational level, who are single parents and not active in the labour market. Most of these women have mixed foreign ethnic origins. It is anticipated that GC can make a difference for these women, yet the current health care model hinders its implementation. The results of the GC_1000 project will be used for advocacy activities targeting policy-makers and health care managers to ensure sustainability of the model.
South Africa	South Africa is of the most unequal countries in the world, reporting a per-capita expenditure Gini coefficient of 0.65 in 2015. Despite free primary health care, including ANC, stark inequities persist between rural and urban areas as well as the private and public health care sectors. Pregnancy is a critical time for diagnosis, maternal treatment and prevention of HIV transmission to children. HIV prevalence rates are as high as 30% amongst pregnant women. In addition, there are clear evidence-based links between alcohol use and health issues, HIV/AIDs and gender-based violence, as well as crime, road accidents and interpersonal violence. Non-, late and infrequent attendance at ANC is amongst the top five avoidable factors in perinatal deaths and amongst the most common underlying causes of patient-related maternal mortality. It is expected that antenatal GC can contribute significantly to tackle these issues.

To allow systematic and consistent identification of the interplay between intervention characteristics and the context in which the intervention is implemented, we chose the Consolidated Framework for Implementation Research (CFIR) as the *basic analytical framework* guiding the GC_1000 project [35]. The CFIR was developed to guide systematic assessment of multilevel implementation contexts and to identify factors that might influence intervention implementation and effectiveness. The CFIR describes five interacting domains for studying implementation and capturing learning [35]. These are:

- The intervention: The characteristics of core components of the intervention, such as complexity, cost and evidence strength, play a crucial role.
- Outer setting: The economic, political and social contexts in which an intervention is carried out and that are external to the implementing organization/institution.
- Inner setting: The context within the implementing organization/institution, including the structure of the organization, its culture (internal climate) and networks and its readiness for change.
- Individuals involved: The characteristics of the people who will have a direct role in the implementation process. This includes educators, health professionals, managers in various parts of the organization/institution, policymakers, service users and many other stakeholders and beneficiaries.
- Process for implementation: This incorporates all methods and approaches used in facilitating, adopting, implementing and continuing the intervention at all levels of the organization, including the planning of strategies and activities. Processes include both those explicitly planned as well as unforeseen processes that emerge during implementation.

Throughout GC_1000, we examine which constructs listed in the CFIR may influence the implementation of GC and consequently implementation outcomes. This will enable us to develop theory-based adaptation and implementation strategies for GC. The methods/methodologies that are used in the different steps are detailed below.

**Fig. 2:**
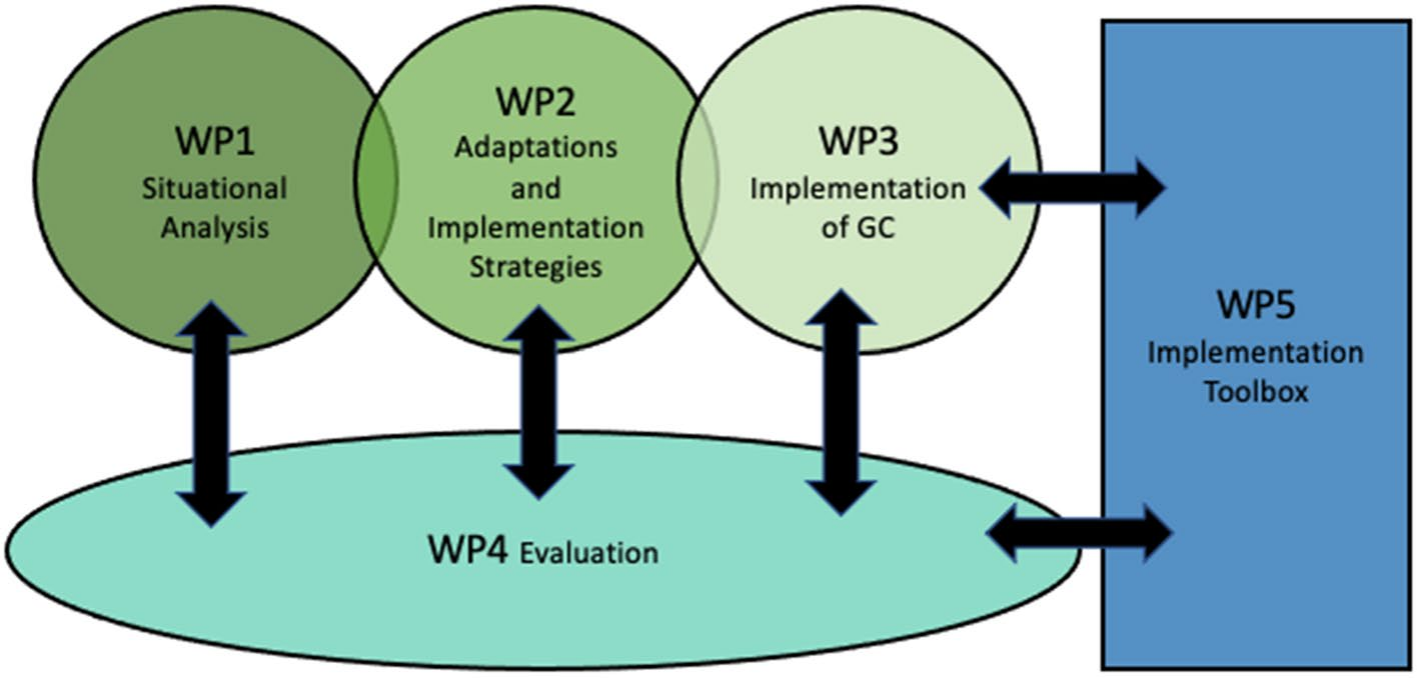
GC_1000 Work Packages

A multi-phase sequential design to implementation has been adopted to achieve our objectives. The GC_1000 consortium is grouped into five inter-related work packages (WPs) with specific tasks, objectives and deliverables, as seen in Fig. 2 (and on the website: https://groupcare1000.com/).

*WP1* leads situational analyses in each setting, with the aim of identifying setting-specific implementation barriers, facilitators and service users’ needs by means of Rapid Qualitative Inquiries (RQI). RQI is a team-based technique for collecting qualitative data in a concise and time-effective way. It is based upon three basic principles:

- Focus on insider’s perspective
- Multiple sources for data collection
- Iterative data collection and analysis allowing for quick preliminary insights [38, 39].

Within RQI, an interdisciplinary team of local and external researchers collects data at the implementation site for a short period of time (approximately 1 week) using multiple methods. For the GC_1000 situational analyses, data were collected using semi-structured interviews and focus groups with providers and recipients of GC and other relevant stakeholders (e.g. policy-makers, community leaders), document analysis and surveys. Iterative adjustment of the data collection strategy occurs in frequent meetings where the collected data are pre-analysed. This procedure enables tailoring of the further data collection (e.g. add questions to topic guide, contact more participants). Research tools and qualitative data analysis will draw on the CFIR [35], allowing for comparison of findings from different sites/countries which will eventually enable the development of blueprints in WP5.

Preliminary findings of the RQIs will be used by *WP2* for the development of tailored implementation strategies and adaptations to the GC model. For this purpose, the cultural sensitivity model will be employed [40]. It distinguishes between surface and deep structure adaptations. Surface adaptation involves matching programme materials and messages to the characteristics of the target populations ensuring cultural sensitivity and responsiveness. Deep structure adaptations stimulate the effectiveness of the intervention by incorporating elements that influence the behaviour of participants in and beneficiaries of the intervention, such as cultural, social and environmental aspects. For the process of adaptation, core questions include when and how to adapt the intervention and which stakeholders to involve in the process [41, 42]. In line with our participatory approach, we will work in close collaboration with women, their partners and families, health care professionals and other stakeholders in the community as well as health systems to adapt GC.

*WP3* will lead GC model implementation, incorporating adaptations formulated in WP2. Implementation success will be fostered at the clinic and country level through intensive training and ongoing interactive support for clinic managers, GC coordinators and GC facilitators. Other experiences have shown that GC implementation can be more effective and efficient when interactive support is provided [43]. Interactive support draws on the Model for Improvement [44], hence helping clinics resolve emerging challenges through continuous planning, monitoring, feedback and adaptations.

Direct support to site teams will be provided by a highly experienced team working around the world over time to implement GC. Support includes training health care providers’ teams to facilitate groups (rather than provide information in a didactic manner), offer basic clinical care within the group setting and show participants how to take and document their own basic health measurements, such as blood pressure and weight. The WP3 team will offer ongoing communication with trainees to answer questions and strengthen their capacity to hold groups and working with local stakeholders to address health system and other administration barriers and to build sustainable programmes. WP3 will offer tools and support materials that will highlight training content and will allow new GC facilitators to carry out groups using interactive adult-learning based methodologies. WP3 also will assist sites to establish their own Steering Committees, including care-providers, support staff, management and client representatives, and where relevant local policy-makers. The Steering Committee is key to local start up and sustainability as members represent different sectors that can either facilitate or provide barriers to GC implementation and sustainability.

**Fig. 3:**
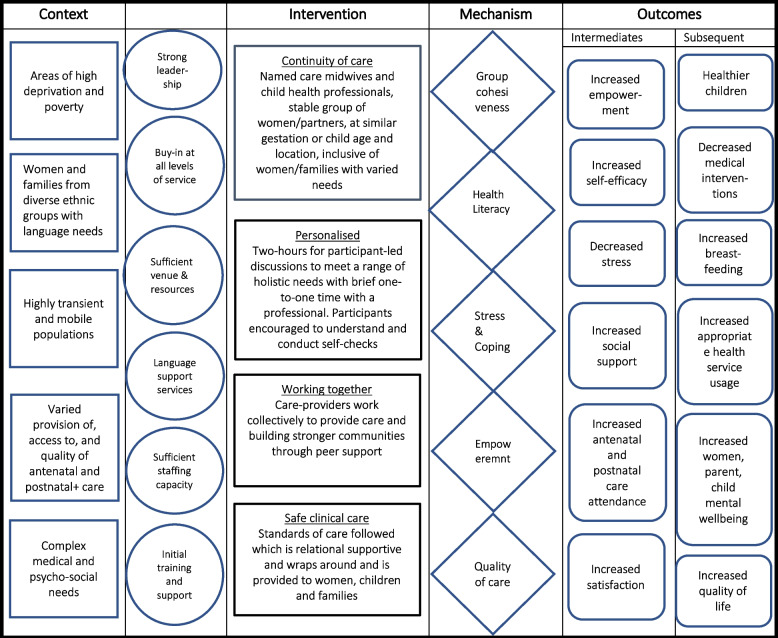
Logic model of context, intervention, mechanism and outcome propositions (from Wiggins et al. 2020)

*WP4* is responsible for the evaluation of process and cost-effectiveness. The process evaluation will be guided by ‘Realistic Evaluation’ principles [45]. Realist evaluation is a theory-based evaluation approach that takes into account the high level of complexity and the role of context in introducing healthcare programmes into dynamic real-world healthcare systems [46]. Rooted in critical realism, it has an explanatory focus that aims to understand how the implementation of programmes are shaped, enabled and constrained by the interaction between programme elements (e.g. organizational changes or interventions) and mechanisms of effect in a diverse range of contexts. A realist evaluation framework is particularly suitable for the evaluation of complex interventions where it is vital to understand how both the context of implementation and the actors involved (including healthcare providers and users) may influence implementation. Data are gathered across all phases of work and analysis at each stage will be synthesized to develop Context-Intervention-Mechanism-Outcome configurations to understand ‘what works, for whom, and in what circumstances’. Figure 3 describes the logic model of context, intervention, mechanism and outcome propositions that will be examined in this evaluation. The model is derived from the prior work of (author and colleagues) in development of the Pregnancy Circles trial in the UK [47].

For the evaluation of the overall programme, we will use an interpretative case study design. Based on data collected during the RQI and the development of adaptation and implementation strategies for GC, we will formulate hypotheses for what GC model and implementation strategies may work, for whom, how, in what circumstances. Additionally, implementation processes and participants’ experiences will be studied by means of observations, surveys, as well as interviews and focus groups with service users and providers. Using these data on implementation processes and participants’ experiences, combined with child and maternal outcome data, we will examine the fidelity and impact of the implementation in the different settings and test the formulated implementation hypotheses.

Process data collection will also include items to enable an estimation of the costs and economic implications of implementing this model in a range of income-level settings as defined by the Organisation for Economic Collaboration and Development (OECD), within varied health systems. Furthermore, an exploratory economic evaluation will be performed in which costs and effects of GC will be compared to usual care using a decision model. Estimates of costs and effects for both forms of prenatal care will be obtained using routine data and data collected by surveys to women receiving GC and women receiving standard care, complemented by information collected in the other WPs, data from literature and expert opinions

*WP5* will develop blueprints for scaling-up GC in each setting, as well as an implementation strategy toolbox. A co-creation approach will be used to translate findings to country-specific blue prints for scaling up GC and developing an implementation strategy toolbox. We will use a time-limited participatory process in which people are brought together to collectively produce an outcome, in this case the blue prints and implementation toolbox for GC_1000. We will set-up multi-stakeholder workshops in each participating country and after the implementation process, we will co-create plans focused on scaling-up GC to other sites and nationwide. As no single stakeholder in antenatal and postnatal care has sufficient expertise or perspective to organize the scaling-up of GC, a multi-stakeholder workshop can help them to think along the same lines and develop innovative approaches that can support further dissemination and buy-in from decision makers. Such workshops are also valuable to influence coordination and commitment to scaling-up and it can help with the integration of local or end-user interests and needs into the scaling-up [48].

Each country will set up a country team consisting of researchers and health care providers who will monitor and support the implementation of GC nationwide. National stakeholder engagement groups will be created to guide and advise the country team on implementation and scaling-up of GC. These stakeholder engagement groups may consist of client representatives, care-providers, researchers, health system administrators and policy-makers, amongst others. Lastly, an international advisory board with scientific experts in antenatal and postnatal care, health inequities and implementation research will be asked to provide advice and guidance throughout the project on study design, analyses, findings and resulting implementation products.

### Data analysis

Data analyses from all stages will be integrated through interpretive synthesis (WP4). To allow for systematic and consistent identification of the interplay between intervention characteristics and the context in which they are implemented, the basic analytical framework for the realist evaluation analysis will be guided by the CFIR. We will examine which factors of the CFIR may influence the implementation of GC and in turn implementation outcomes, framing this analysis within the Context, Intervention, Mechanism, Outcome (CIMO) configurations characteristic of realist evaluation. Data analysis will initially be inductive but will be mapped to these components and then synthesized with outcome data using CIMO configurations. The data analysis from WP1 will form the basis for the Context component of the realist evaluation, while the analysis from WP2 will form the basis for the Intervention (implementation strategy) and Mechanism components and the analysis from WP4 will synthesize all these elements also in relation to the outcome component of the realist evaluation.

This will allow us to assess what works for whom, in what circumstances.

Qualitative data of the WPs will be analysed inductively initially by applying open coding and thematic analysis, using qualitative data analysis software. Following the initial coding and identification of candidate themes, these will be mapped onto the CFIR framework. Any themes that do not fit the CFIR will be identified, and the framework adjusted if appropriate.

Quantitative data will be imported into SPSS files. The primary data analysis will be descriptive. Secondary inferential analyses will be conducted to identify possible indicators of impact as follows:

- Pre- and post-implementation routine outcomes data and process data
- Data for those in GC compared with existing local, regional or national data

As it will not be possible to provide matched controls or a controlled comparison group in this study, statistical adjustments may be used to control for any socio-demographic, ethnic or clinical differences between women receiving GC and the local, regional or national reference population.

Research findings from the GC_1000 project will adhere to reporting standards for qualitative research, following the 32-item checklist for interviews and focus groups (COREQ) [49] and the 22-item checklist for reporting observational research (STROBE) [50].

## Discussion

GC is an innovative care model to provide antenatal and postnatal+ care holistically, in a group format. Despite promising findings, the GC model has not yet been successfully disseminated and integrated into standard maternal and child health care in settings with relatively high rates of adverse neonatal and maternal outcomes. Disparate factors are likely to influence the implementation of GC in diverse health care systems. Within GC_1000, we will study the implementation of GC systematically, generating evidence that will enhance the current knowledge base about the integration of complex interventions into established health care settings.

### Strengths and limitations

A strength of the GC_1000 study design lies in the application of realist evaluation principles. Instead of exclusively focusing on outcomes, this study seeks to explain which implementation mechanisms are at play in what context and why they may interactively lead to certain outcomes. Moreover, the development of research tools is informed by the CFIR and it hence is theory-driven. In this way, GC_1000 contributes to the reduction of the prevailing lack of theory-informed implementation research in maternity care [5, 11].

It is crucial to involve relevant stakeholders in implementation projects from the beginning to adapt interventions and implementation strategies according to their needs. At the core of the GC_1000 design lies a participatory approach where relevant stakeholders are involved and facilitate sustained implementation and scale-up. As such, country teams will document all their activities and discussions as well as relevant developments within the country, adding to the rich variety of data.

A further strength of the study design is triangulation at multiple levels. Methodological triangulation is achieved through the use of qualitative and quantitative methods to investigate the same phenomenon. Aiming for a rich and broad understanding of implementation processes and outcomes, data will be collected from various sources and respondent categories. The generated data will then be interpreted by our multidisciplinary team of local and external researchers which will shed light from various perspectives on our findings. This integration of emic and etic perspectives is aimed at reducing ethnocentrism as much as possible.

However, our study design is not free from limitations which we aim to counter in various ways. For instance, most measurements will rely upon self-report data which are prone to memory and social desirability biases and the composition of our sample may be affected by selection bias. We hence make use of triangulation to minimize the impact of such biases.

Whereas member checking of findings with interviewees will be limited due to logistical challenges and the large amount of qualitative data that will be generated, summaries of preliminary findings will be discussed within the local research teams. Considering the relatively large number of researchers who will conduct interviews and focus groups, it will also not be possible to acknowledge how researchers influence narratives; thus, reflexivity will be contingent. However, each member of the research team will keep a research diary throughout the process, documenting reflective notes.

As this programme is primarily focused on understanding the implementation process, with adaptations to and led by each local setting, the study does not include a matched or randomized control group. However, where feasible, we intend to include data from comparable settings or from the same sites prior to implementation of GC. Where relevant, statistical adjustments will be used to control for any socio-demographic or clinical differences between women receiving GC and the reference population. The outcome data for those in GC will also be considering within the context of existing local, regional or national data. We consider outcome data as indicators of implementation fidelity and effectiveness, rather than as formal clinical outcome measures, as the study aims are focused primarily on understanding implementation challenges, successes and adaptation to context.

Lastly, the Covid-19 pandemic poses a multitude of challenges to research and implementation processes. As such, our data collection methods, and the GC model itself, might need adaptations. If external researchers may not be able to travel to the implementation sites, online interviews and other virtual data collection methods need to be employed. Data collection may also rely more heavily on local research teams due to travel restrictions. However, such adaptations depend on the specific situations in each country and situations might vary significantly with regard to coping of the health care system with the pandemic, but also with regard to availability of online research tools.

### Implications

GC_1000 findings and tools will be widely disseminated and they have the potential of multi-level impact:

- A better understanding of implementation and scaling-up processes with regard to different contexts and resource requirements
- Information on how to initiate, support and achieve sustainability
- Prevention of adverse health outcomes for mothers and their babies as well as behaviour changes that lead to healthy lifestyle choices and improved health literacy and parenting skills
- Improved satisfaction with care, both on the part of participants and health care facilitators
- Methods to calculate the costs and benefits of the implementation of GC in diverse settings

This 4-year project will generate evidence-based knowledge about the integration of complex interventions in diverse health care systems and also will pave the way for sustained implementation of GC, with special attention to mothers, families and communities who can benefit most.

## Data Availability

Not applicable.

## Abbreviations

GC_1000: Group Care during the first 1000 days
MMR: Maternal mortality ratio
LMIC: Low- and middle-income countries
GC: Group Care
CBGC: Centering-based Group Care
CIMO: Context-Intervention-Mechanism-Outcome
ANC: Antenatal care
PNC: Postnatal care
HIV: Human immunodeficiency virus
AIDS: Acquired immunodeficiency syndrome
RQI: Rapid Qualitative Inquiries
CFIR: Consolidated Framework for Implementation Research
OECD: Organisation for Economic Collaboration and Development
WP: Work Package
COREQ: COnsolidated criteria for REporting Qualitative research
STROBE: Strengthening the Reporting of OBservational studies in Epidemiology

## Footnotes

1 For the purpose of this research, de Groot and colleagues’ definition of vulnerability [3] was adapted to: ‘Vulnerability is a dynamic state that reflects converging effects of a set of interacting and amplifying personal, environmental and structural factors, where risk factors outweigh protective factors leading to enhanced susceptibility to adverse health outcomes in the first 1000 days and hampering recovery.’
